# Respiratory Viral Infections and Subversion of Cellular Antioxidant Defenses

**DOI:** 10.4172/2153-0645.1000141

**Published:** 2014-09-30

**Authors:** Narayana Komaravelli, Antonella Casola

**Affiliations:** 1 Department of Pediatrics, University of Texas Medical Branch, Galveston, TX, USA; 2 Department of Microbiology and Immunology, University of Texas Medical Branch, Galveston, TX, USA; 3 Department of Sealy Center for Vaccine Development, University of Texas Medical Branch, Galveston, TX, USA

**Keywords:** Respiratory syncytial virus, Oxidative stress, Nrf2, ROS, Free radicals

## Abstract

Reactive oxygen species (ROS) formation is part of normal cellular aerobic metabolism, due to respiration and oxidation of nutrients in order to generate energy. Low levels of ROS are involved in cellular signaling and are well controlled by the cellular antioxidant defense system. Elevated levels of ROS generation due to pollutants, toxins and radiation exposure, as well as infections, are associated with oxidative stress causing cellular damage. Several respiratory viruses, including respiratory syncytial virus (RSV), human metapneumovirus (hMPV) and influenza, induce increased ROS formation, both intracellularly and as a result of increased inflammatory cell recruitment at the site of infection. They also reduce antioxidant enzyme (AOE) levels and/or activity, leading to unbalanced oxidative-antioxidant status and subsequent oxidative cell damage. Expression of several AOE is controlled by the activation of the nuclear transcription factor NF-E2-related factor 2 (Nrf2), through binding to the antioxidant responsive element (ARE) present in the AOE gene promoters. While exposure to several pro-oxidant stimuli usually leads to Nrf2 activation and upregulation of AOE expression, respiratory viral infections are associated with inhibition of AOE expression/activity, which in the case of RSV and hMPV is associated with reduced Nrf2 nuclear localization, decreased cellular levels and reduced ARE-dependent gene transcription. Therefore, administration of antioxidant mimetics or Nrf2 inducers represents potential viable therapeutic approaches to viral-induced diseases, such as respiratory infections and other infections associated with decreased cellular antioxidant capacity.

## Introduction

Molecular oxygen is essential for supporting the life processes of all aerobic organisms. Under physiological conditions, oxygen is combusted in a highly controlled manner by the cell’s metabolic machinery to obtain chemical energy in form of ATP, and this process leads to the formation of reactive oxygen species (ROS) [1,2]. ROS are unstable molecules, which in small quantities are involved in cellular signaling, but become toxic when produced in large quantities by initiating oxidation of cellular components such as proteins, lipids, and DNA [1]. ROS are broadly classified into two groups, radical and non-radicals. Members of the radical group, often called free-radicals, have at least one unpaired electron in the outer orbital and therefore are highly reactive, as they readily donate or accept an additional electron to achieve stability [3,4]. This group includes compounds such as superoxide ion radical (O^·^_2_^−^), hydroxyl radical (OH^·^), nitric oxide radical (NO^·^), peroxyl (ROO^·^) and alkoxyl radicals (RO^·^) [1,5,6]. The non-radicals group includes compounds such as hypochlorous acid (HClO), hydrogen peroxide (H_2_O_2_), organic peroxides and aldehydes. In addition to endogenous ROS, exogenous compounds such as air pollutants, cigarette smoke, radiation, heavy metals etc., can generate ROS [7]. In order to protect from the continuous exposure of exogenous and endogenous ROS, organisms have developed a complex antioxidant system which include enzymatic (superoxide dismutase, catalase, glutathione peroxidase, etc.) and non-enzymatic (transferrin, ferritin, vitamin A and C, etc.) defenses. Failure to keep the equilibrium between ROS formation and antioxidant defenses leads to oxidative stress. This is characterized by an augmented generation of oxidant species and reduced antioxidant cellular capacity [2,8–11]. At molecular level, the oxidative damage to DNA cause polysaccharide ring cleavage, base modification or chain breakage, leading to mutations and altered/ failed gene transcription; damage to proteins can modify functional groups, such as addition of nitro radicals and carbonyl groups, resulting in altered activity, aggregation, fragmentation and/or cleavage; damage to lipids leads to formation of lipid aldehydes, lipid peroxides, causing changes in fluidity and permeability of membranes [6,12,13].

While ample information is available about the mechanism(s) of increased ROS generation, little is known about the regulating changes in antioxidant enzymes (AOE) expression [8]. At the gene expression level, many of the genes coding for AOE are controlled by the redox sensitive transcription factor NF-E2-related factor 2 (Nrf2), binding to promoter antioxidant responsive element (ARE) sites. These ARE elements are also present in the regulatory regions of many genes encoding phase-2 detoxification enzymes and various cytoprotective proteins, such as NAD(P)H:Quinoneoxidoreductase (NQO1) [14–16]. Nrf2 is a cap’n collar basic leucine-zipper transcription factor, which under normal physiologic conditions is sequestered in the cytoplasm by Kelch-like ECH associated protein 1 (Keap1), forming a complex bound to the cytoplasmic membrane through actin [17,18]. In the presence of elevated levels of ROS and cellular oxidative stress, Nrf2 is released from this complex by conformational change in cysteine disulfide bonds of Keap1 [19–22]. Nrf2 is then phosphorylated at serine 40 by protein kinase C and translocate to the nucleus [23], where it forms DNA-protein complexes with transcription factors belonging to the small *m*usculo*a*poneurotic *f*ibrosarcoma (Maf) and transcriptional co-activators, such as CREB binding protein (CBP) and p300, to initiate transcription of ARE-dependent genes [17]. Once the cellular redox status returns to equilibrium, Keap1 sequesters Nrf2 and directs it to Cul3-ubiquitin mediated proteasome degradation [17,21]. In the past few years, the Nrf2-Keap1/ARE system has been the focus of intense investigation, because of it possible role in the pathogenesis of several diseases [16,24].

Generation of oxidative stress has been reported in about 200 diseases [24] and oxidative stress is thought to play an important pathogenic role in pulmonary disorders such as chronic obstructive pulmonary disease (COPD) [25] and asthma [26–28], cancer [29,30], neurological diseases, including Alzheimer’s [24,31], cardiovascular [32] and metabolic disorders, such as diabetes [33], vision disorders [34] and aging [35]. This review focus on Nrf2 and oxidative stress associated with respiratory viral infections with emphasis on respiratory syncytial virus (RSV), although other viruses, including human immunodeficiency virus (HIV), Hepatitis B and C have been shown to induce ROS *in vitro* and *in vivo* [36–39]. Free radicals generated during various respiratory viral infections are showed in Table 1.

### Respiratory syncytial virus

RSV is an enveloped, negative-sense, single-stranded RNA virus belonging to *Paramyxoviridae* family, and is the leading cause of respiratory diseases in infants and young children. Annually in the US alone, RSV infections are responsible for more than 100,000 hospitalizations among children <1 year of age and accounts for ~1.5 million outpatient visits among children <5 years of age, with economic burden of more than $500 million/year [40–42]. Worldwide each year, an estimated 33.8 million new episodes of RSV-associated acute lower respiratory tract infections (ALRI) occur in children <5 years of age, with about 3.4 million children requiring hospital admission, and an estimated 66,000–199,000 fatal case, mostly in developing countries [43]. RSV infection is also a major concern in elderly people with chronic heart and lung diseases, and in immunocompromised patients [44]. Around 1500 to 7000 deaths due to RSV infection occur in the USA alone each year, especially in people >65 years of age [45]. Although RSV has been the focus of intense investigation for several decades, no effective drug or vaccine is currently available [46]. While the mechanisms of RSV-induced airway disease and its associated long-term consequences are not fully understood, lung inflammatory response and oxidative stress are important pathophysiological features of RSV lower respiratory tract infections [47]. This review focuses on the potential role of oxidative lung damage in RSV pathogenesis and possible novel therapeutic approaches targeting ROS formation and Nrf2 activation in the context of this, as well as other respiratory viral infections.

### ROS generation in RSV infection

As mentioned before, ROS formation occurs as part of aerobic cellular metabolism and plays an important role in cellular signaling, leading to the expression of a variety of molecules, including proinflammatory mediators, such as cytokines and chemokines [48]. If ROS are not neutralized by cellular antioxidant systems, they can cause extensive cellular and tissue damage. Many of the features of acute and chronic lung diseases, such as bronchoconstriction, airway hyper reactivity, enhanced mucous secretion, epithelial cell damage, and microvascular leakage, have been shown to be associated with oxidative stress due to increased generation of ROS [28]. RSV infection has been reported to enhance ROS formation in airway epithelial cells, the primary target of infection, as measured by the fluorescent probe 2′,7′ dichlorodihydrofluorescein diacetate [49–52]. RSV infection leads to the release of superoxide, H_2_O_2_and myeloperoxidase (MPO) in the extracellular environment by inducing the recruitment and activation of neutrophils and eosinophils into the airways [53,54]. ROS generation in airway epithelial cells during RSV infection was recently summarized in a review by Garofalo et al. [47]. Several NADPH oxidase inhibitors, including diphenyleneiodonium chloride (DPI), apocynin, and 4-(2-aminoethyl) benzene sulfonyl fluoride (AEBSF), inhibit RSV-induced cellular signaling in airway epithelial cells, in particular chemokine expression, as well as activation of the transcription factors interferon regulatory factor-3 (IRF-3), signal transducer and activator of transcription (STAT)-1 and the upstream kinase inhibitor of κB kinase epsilon (IKKε) [55–58].

In regard to other respiratory viruses, rhinovirus infection has been associated with superoxide and hydrogen peroxide production through NOX1 [59] and influenza infection generates superoxide through NOX2 [60], as shown in Table 1.

### Oxidative stress in RSV infection

RSV-induced ROS formation is associated with significant cellular oxidative stress *in vitro* as well as *in vivo*, as a result of disruption of the fine balance between pro-oxidant and antioxidant factors. During RSV infection of airway epithelial cells, SOD 2 expression and activity progressively increases, with a progressive decrease in the expression of all the other tested AOEs such as SOD 1, SOD 3, catalase, GST expression, and GPx activity. These changes in AOE expression suggest that increased amounts of superoxide, generated by RSV through NADPH oxidase, could result in accumulation of H_2_O_2_ by increased SOD 2 activity and reduced activity of catalase, GST and GPx [8]. The non-detoxified H_2_O_2_, as well as other radicals generated from H_2_O_2_ auto-oxidation in presence of transition metals, such as the hydroxyl radical (^·^OH), reacts with lipids, proteins and DNA, causing structural cellular damage. Proteomics studies have also shown that several AOEs, from peroxiredoxins to catalase, SOD 1, GPx 1 and various forms of GST, are significantly decreased in the lungs of infected animals compared to uninfected (Table 2 summarizes all antioxidant proteins whose expression in broncho alveolar lavage (BAL) changes in response to RSV infection). Decreased expression/activity of antioxidant proteins was confirmed *in vivo*, both in a mouse model of RSV infection as well as in children with severe bronchiolitis [11].

Lipid peroxidation refers to the oxidative degradation of lipids. It is the process in which free radicals “steal” electrons from the lipids in cell membranes, resulting in cell damage. It most often affects polyunsaturated fatty acids. The end products of lipid peroxidation are reactive aldehydes, such as malondialdehyde (MDA) and 4-hydroxynonenal (HNE), the second one being known also as “second messenger of free radicals” and major bioactive marker of lipid peroxidation, due to its numerous biological activities resembling activities of reactive oxygen species. In addition to MDA and HNE, 8-isoprostane are also considered markers of cellular oxidative stress, as they are formed *in vivo* from the free radical-catalyzed peroxidation of essential fatty acids (primarily arachidonic acid) without the direct action of cyclooxygenase enzymes. The unbalance between ROS formation and antioxidant defenses leads to oxidative stress during the course of RSV infection, as it has been demonstrated by the increased formation of lipid peroxidation products both *in vitro* and *in vivo* models of infection [8,61], as well as in patients with primary RSV infection, in which the levels of 8-isoprostane, as well as MDA and HNE present in respiratory secretion correlate with the severity of infection [11].

In addition to RSV, the closely related human metapneumovirus (hMPV), which is also a common cause of lower respiratory tract infections in children [62], significantly affects AOE expression *in vitro* and *in vivo*. Microarray analysis of gene expression studies from hMPV infected airway epithelial cells demonstrated progressively decreased levels of SOD 3, catalase, GST and peroxiredoxin gene expression and protein levels, with a concomitant increase in SOD 2 [63], similar to what has been observed with RSV. These changes in AOE expression was also observed in a mouse model of hMPV infection [11]. Such an increase in SOD 2 expression and decreased expression of catalase, as well as decreased GSH/GSSG ratio, has also been reported in influenza infection both *in vitro* and *in vivo* [64–66]. Taken together, this information suggests that airway oxidant-antioxidant imbalance could play a very important role in the pathogenesis of RSV-induced lung disease and possibly other respiratory viral infections.

### Potential regulatory mechanism of AOE expression in RSV infection

**Figure 1:**
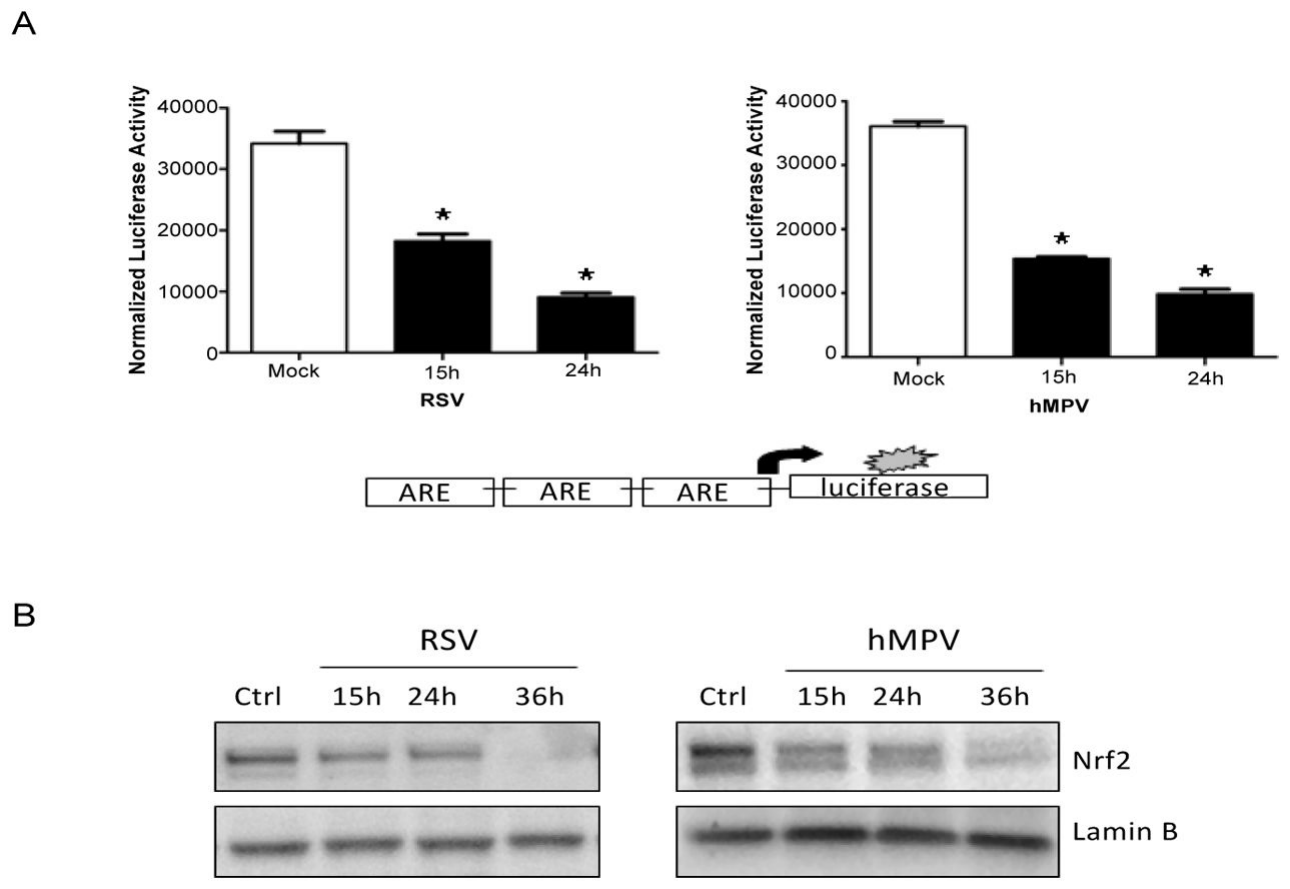
RSV and hMPV infection modulates ARE-dependent gene transcription (A) A549 cells were transiently transfected with a plasmid containing multiple copies of the NQO1 ARE site linked to the luciferase gene and then infected with either RSV (Left panel) or hMPV (Right panel). Cells were harvested at different times post-infection to measure luciferase activity. Uninfected cells, transfected with reporter plasmid only and mock-infected, served as controls. For each plate luciferase was normalized to the β-galactosidase reporter activity. Data are expressed as mean ± standard deviation of normalized luciferase activity. *P <0.05 relative to RSV or hMPV infected cells. (B) Nuclear extracts prepared from A549 cells infected with RSV (left panel) or hMPV (right panel) for various periods of time post infection (p.i.) were subjected to western blot with anti Nrf2 antibody. Membranes were stripped and reprobed for lamin B as an internal control for protein integrity and loading.

**Figure 2:**
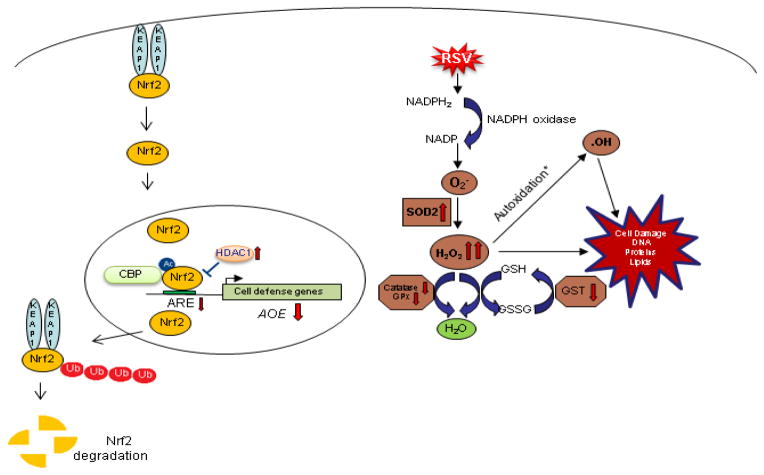
Schematic representation of the proposed mechanisms of oxidative cell damage during RSV infection RSV infection of airway epithelial cells leads to increased superoxide formation and increased H_2_O_2_ production, due to up regulation of SOD 2 expression and activity. RSV-induced inhibition of Nrf2 activation, due to proteasome-dependent degradation, causes a progressive decrease in the expression of a variety of AOEs involved in H_2_O_2_ detoxification leading to accumulation of highly reactive radicals, such as hydroxyl radical, and subsequent cellular damage (* autoxidation in presence of transition metals).

The exact mechanism of decreased expression of AOEs during RSV infection, as well in the context of other viral respiratory infections, is largely unknown. Most of the AOE gene expression is regulated in part through ARE sequences and Nrf2 activity [30,67]. Transcription factor Nrf2 is an important redox-responsive protein that protects the cells from oxidative stress and injury (Reviewed in [16]). Nrf2-dependent AOE gene expression might be reduced by: (i) competition for binding to the ARE site - Bach1/small Maf protein complex or AP-1 family transcription factors like c-Fos and FRA1 can bind to ARE acting as a transcriptional repressor [67,68]; (ii) preventing Nrf2 activation through direct physical association - Activating transcription factor (ATF)3 or retinoic acid receptor α were shown to form inhibitory complexes with Nrf2, leading to displacement from ARE elements; (iii) interfering with recruitment of co activators, such as CBP, to the ARE site - NF-κB activation can lead to decreased availability of CBP and promote the recruitment of co repressors (histone deacetylases) at Nrf2-ARE site [67]; (iv) reduced nuclear levels, which can occur due to enhanced nuclear to cytoplasm efflux or increased Nrf2 degradation [8]. A recent study has shown that RSV infection in *Nrf2−/−* mice is more severe and associated with higher viral titers, augmented inflammation, enhanced mucus production and epithelial injury compared to Nrf2 wild type mice, indicating the protective role of Nrf2-ARE pathway against RSV infection [69]. RSV infection can indeed induced a progressive decrease in ARE-dependent gene transcription in A549 cells, carcinoma-derived type II-like airway epithelial cells, investigated using luciferase reporter gene assays (Figure 1A, left panel)[47]. A similar result was obtained when cells were infected with hMPV (Figure 1A, right panel). Reduced nuclear levels of Nrf2 was observed in both RSV infection (Figure 1B, left panel) [8] and hMPV infection (Figure 1B, right panel), together with increased nuclear levels of known ARE transcriptional repressors such as Bach1 and ATF3 (Casola A, unpublished observation) [47], suggesting a potential mechanism for viral-induced down regulation of AOE gene expression. As Nrf2 positively regulates its own gene transcription, reduced Nrf2 mRNA levels were observed in airway epithelial cells at late time point of RSV infection [8]. Our recent studies indicate that RSV infection is associated with Nrf2 deacetylation, likely due to RSV-induced upregulation of histone deacetylase (HDAC) activity, and increased degradation, which occurs through the ubiquitin-proteasome pathway. Blocking proteasome and class I HDAC activity, in particular HDAC 1 and 2, rescued Nrf2 activation and ARE-dependent gene expression during RSV infection (Casola A, unpublished observation). A summary of findings and a proposed model of RSV-induced oxidative stress in airway epithelial cells are depicted in Figure 2.

In regard to other respiratory infections, Yageta et al. showed that influenza infection in Nrf2-deficient mice is associated with increased mortality, compared to wild type mice, when animal are exposed to cigarette smoke. Nrf2-deficient mice could not control the oxidative stress caused by cigarette smoke and showed enhanced peribronchial inflammation, lung permeability damage, and increased mucus secretion [70]. Inhibition of Nrf2-dependent gene expression in differentiated human nasal epithelial leads to increased influenza virus entry and replication, due to increased oxidative stress [39]. In addition, overexpression of Nrf2 in alveolar type II cells provides protection from influenza infection by reducing oxidative stress and viral replication [71]. These data suggest that Nrf2 plays an important role in influenza infection by controlling ROS formation, viral replication and lung injury.

### Therapeutic approaches

Since oxidative stress seems to play an important role in the pathogenesis of RSV, and possibly other viral-associated lung diseases, antioxidant intervention would represent a rational approach for treatment of lower respiratory tract infections. Table 3 shows antioxidant therapies tested against various respiratory viral infections. Antioxidants reduce oxidative stress by quenching free radicals and help the host to function properly. Our group has tested two complementary approaches that can affect the outcome of RSV-associated lower respiratory tract infections: (i) SOD mimetics that can scavenge free radicals and reduce oxidative stress in RSV infected cells and; (ii) induction of airway antioxidant defenses by modulating AOE gene expression/activity.

#### SOD mimetics

**Figure 3:**
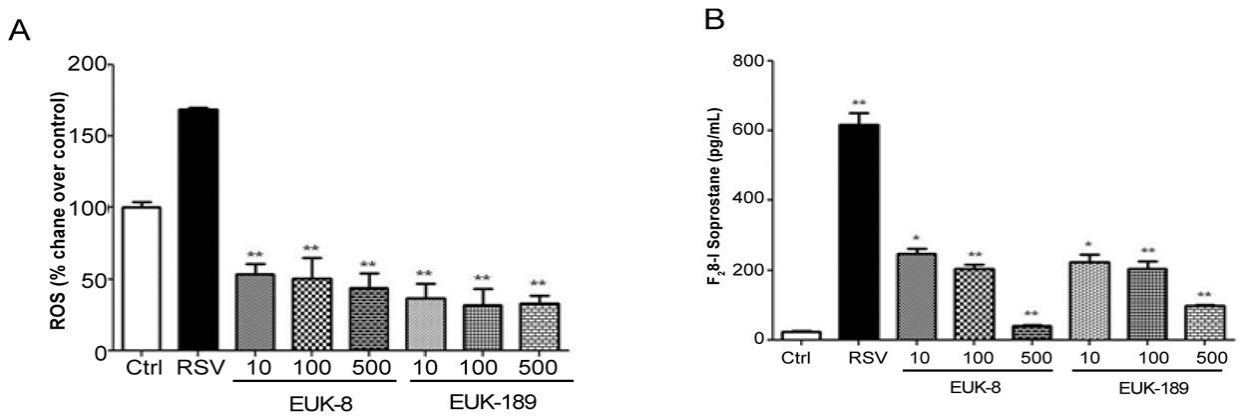
Effect of EUK treatment on RSV-induced ROS formation and oxidative stress (A) A549 cells were treated with different micromolar concentrations of EUK-8 and EUK-189, infected with RSV for 24 h, and harvested to measure DCF-DA fluorescence. Ctrl indicates uninfected cells. Mean Fluorescence Intensity is reported as percent increase over control. (B) Cell supernatants were harvested at 24 h p.i. to measure F2-isoprostanes. Results are expressed as mean ± standard error. Results are representative of two independent experiments run in triplicate. *p<0.05, **p<0.01 compared to untreated RSV-infected cells. Reprinted with permission of the American Physiological Society. Copyright © 2014 The American Physiological Society [78].

SOD 1 and 2 administration and SOD 3 overexpression have been shown to protect mice lungs from influenza-induced oxidative stress damage [72]. Both SOD 1 and 2 administration, either parenterally or intranasal in a cotton rat model of RSV infection, reduced pulmonary viral titer [73]. In the past few years, quite a few classes of synthetic SOD mimetics that are based on organo-manganese complexes have been developed and explored as possible therapeutics against oxidant-related lung damage [74]. In a recent study, the effect of airway epithelial cell treatment with various Eukarionsalen-manganese complexes (EUK) on cellular signaling and oxidative stress in response to RSV infection was assessed. EUKs are synthetic salen-manganese complexes that exhibit SOD and catalase activities [75]. Salen complexes are Schiff bases, usually prepared by the condensation of a salicylaldehyde with an amine. Based on the salen ring substitutes, EUKs are named from EUK-8 to EUK-189 and have different rates of SOD and catalase/peroxidase activities [76,77]. Treatment of RSV-infected airway epithelial cells with EUK-8, -134 and -189, which have SOD and catalase/peroxidase activity, results in significantly reduced ROS levels (Figure 3A) and markers of oxidative cell damage (Figure 3B). In addition, EUK treatment is associated with reduced activation of the viral-induced transcription factors NF-κB and IRF-3 and reduced secretion of cytokines and chemokines [8,78]. Enhancement of both SOD and catalase/GPx activities is important to reduce ROS levels and pro-inflammatory gene expression during RSV infection, as EUK-163, which has no significant catalase or peroxidase activity, does not have a significant effect on RSV-induced pro-inflammatory mediator secretion [8]. In addition, EUK treatment in high concentration (500 μM) significantly reduce viral replication [78], suggesting that EUKs could represent a novel therapeutic approach to modulate RSV-induced lung damage.

#### Nrf2 inducers

Several compounds that stimulate Nrf2-ARE driven transcription have been identified from natural and dietary sources, metabolites, and synthetic agents. Nrf2 inducers are broadly divided into Triterpenoids that include oleanolic acid and ursolic acid (natural); oleanane triterpenoids, 2-cyano-3,12-dioxooleana-1,9,-dien-28-oic acid (Synthetic) [79,80]; Isothiocyanates including sulforaphane, found mainly in cruciferous vegetables; polyphenols including flavonoids quercetin and EGCG and the non-flavonoids curcumin, resveratrol and butylated hydroxyanisole (BHA) [81,82].

Sulforaphane modifies a number of cysteine residues in Keap1 through formation of carbamodithioate and releases Nrf2 that leads to increased nuclear localization of Nrf2 and ARE transcription [82]. Sulforaphane pretreated nasal epithelial cells during influenza virus infection showed significantly increased levels of Nrf2 and HO-1 associated with reduced hemagglutinin gene expression and viral replication [39]. In a model of RSV infection, mice treated with sulforaphane showed significantly reduced numbers of neutrophils and eosinophils in BAL after infection [69], suggesting that this compound has the potential for modulating viral-induced oxidative stress and disease.

BHA and t-Butyl hydroquinone (tBHQ) treatment induces phase II enzymes HO-1 and NQO1 *via* Nrf2-ARE transcription in rat and human hepatocytes [83]. Hence, BHA was assessed for its ability to modulate oxidative stress in a mouse model of RSV infection. BHA treatment significantly attenuated RSV-induced lung oxidative stress, as indicated by decreased markers of oxidative damage in BAL of RSV-infected mice. In addition, lungs of BHA treated mice showed reduced cytokine and chemokine secretion [61]. The beneficial effect of BHA and tBHQ in RSV-induced lung inflammation and oxidative stress could be in part ascribed to the ability of these phenolic compounds to modulate Nrf2-dependent gene expression, in addition to directly scavenging ROS formed in response to the viral infection. Preliminary studies revealed that treatment of airway epithelial cells with tBHQ significantly increased ARE-dependent gene transcription and Nrf2 protein expression. tBHQ treatment rescued Nrf2-ARE driven activity during RSV infection and also ameliorated RSV induced oxidative damage as demonstrated by reduced lipid damage (Casola A, unpublished observation).

## Conclusion

Respiratory tract infections are a leading cause of morbidity and mortality worldwide. RSV and other viruses such as influenza and hMPV are a major cause of pediatric upper and lower respiratory tract infections, associated with bronchiolitis, pneumonia and flu-like syndromes, as well as asthma exacerbations. There is still no vaccine or effective treatment available for RSV infections, as well as for many other respiratory viruses, necessitating an explicit understanding of the pathogenic mechanisms associated with these infections. As oxidative stress is likely to play an important role in initiating and sustaining lung injury and inflammation, approaches that combine scavenging ROS together with the inhibition of viral replication, may be effective in modulating severe lung disease associated with RSV and other viral respiratory infections. This could be obtained by either administration of antioxidant compounds that possess antiviral activity, in addition to ROS scavenging properties, or by combining antivirals with compounds capable of increasing lung antioxidant defenses, such as AOE mimetics or Nrf2 inducers. These treatment approaches would be effective only if compounds are available at the site of infection, therefore route of administration, bioavailability, tissue distribution are all important parameters that will need to be taken into consideration when planning future therapeutic intervention.

## Figures and Tables

**Table 1:** Free radicals generated in response to respiratory viral infections.

Virus	Free radical generated	Effect	Host proteins associated with oxidative stress	Ref
Rhinovirus	superoxide (O_2_^−^), hydrogen peroxide (H_2_O_2_)	airway inflammation	activation of xanthine oxidase; reduced concentration of glutathione (GSH) and increased activity of NADPH oxidase 1 (NOX1)	[59,84–87]
Influenza Virus	O_2_^−^, nitric oxide (NO)	enhanced viral mutations, replication and airway inflammation	reduced concentrations of catalase, glutathione and super oxide dismutase (SOD); Increased activity of NADPH oxidase 2 (NOX2)	[60,66,88–91]
RSV	NO, O_2_^−^, H_2_O_2_	airway inflammation	virus induced nitric oxide synthase (iNOS) activity; progressive decrease of antioxidant enzymes SOD 1, SOD 3 and Catalase; reduced nuclear translocation of Nrf2 and Nrf2-ARE driven transcription	[8,11,52,92] (Casola A, unpublished observation)
hMPV	O_2_^−^, H_2_O_2_	airway inflammation	progressive decrease of antioxidant enzymes SOD 3, catalase, GST, and Prdx; reduced nuclear translocation of Nrf2 and Nrf2-ARE driven transcription	[11,63] (Casola A, unpublished observation)

**Table 2:** Differential expression of antioxidant proteins in bronchoalveolar lavage of respiratory syncytial virus-infected mice.

	Fold Change in RSV BAL Compared to Control				
AOE	Day 1	Day 3	Day 5	Day 9	Day 25
1-Cys peroxiredoxin protein	−1.0	−6.1	—	−4.1	—
Catalase	—	−2.5	−2.1	—	—
Cu/Zn SOD 1	−2.3	−3.4	−2.0	−2.0	—
Glutathione peroxidase 1	−1.8	−2.3	—	1.3	—
Glutathione S-transferase	—	—	—	−6.0	—
Glutathione S-transferase omega 1	−6.8	−3.6	−2.3	−2.0	1.3
Glutathione S-transferase, alpha 4	−2.2	—	—	—	—
Glutathione S-transferase, mu 1	—	−4.0	−7.0	−1.7	1.4
Glutathione S-transferase, mu 2	—	−4.3	—	3.4	−1.3
Glutathione-disulfide reductase	—	—	—	3	—
Nonselenium glutathione peroxidase	−2.6	—	−4.2	−1.3	1.2
Peroxiredoxin 6	—	−3.1	−3.9	−4.1	1.3
Peroxiredoxin 2	2.7	2.4	−2.1	1.7	—
Thioredoxin 1	—	1.5	—	—	1.1

Shown are high probability antioxidant protein identifications and their expression (in terms of fold changes in RSV BAL compared to control mice) at different days of p.i. from peptide mass fingerprinting in MALDI-TOF/MS. BAL, bronchoalveolar lavage; —, not determined.

Reprinted with permission of the American Thoracic Society. Copyright © 2014 American Thoracic Society [8,11].

**Table 3:** Antioxidant therapy against respiratory viruses.

Virus	Antioxidant	Effects	Ref
Rhinovirus	Glutathione	Scavenge free radicals and suppress NF-κB induced Intercellular adhesion molecule-1 (ICAM-1) receptor for rhinovirus	[84]
	Pyrrodolinedithiocarbonate (PDTC)	Inhibited viral replication *in vitro*	[93]
Influenza Virus	**Thiol antioxidants** Pyrrodolinedithiocarbonate (PDTC)	Scavenge OH^−^ free radicals and Inhibited synthesis negative strand RNA and viral replication *in vitro* and *in vivo*	[72,94]
	*N*-Acetyl-L-cysteine (NAC)	Inhibited induction of apoptosis and pro-inflammatory cytokines such as IL-6, IL-8 and RANTES	[95,96]
	Glutathione	Inhibited induction of apoptosis and viral replication *in vitro* and *in vivo*	[97,98]
	**Hydroxyl antioxidants/Polyphenols** Nordihydroguaiaretic acid (NDGA)	scavenge O_2_^−^, OH^−^ radicals and H_2_O_2_and Inhibited viral replication through inhibiting intracellular transport of viral glycoproteins	[99–104]
	Thujaplicin	Inhibited induction of apoptosis and viral replication *in vitro*	[100]
	Resveratrol	Blocked nuclear cytoplasmic translocation viral ribonucleoproteins and reduced expression of late viral proteins and resulted in reduced viral replication *in vitro* and *in vivo*	[101]
	Ambroxol	Suppressed proliferation of virus *in vivo*	[103]
	Ascorbic acid	inhibited the proliferation of virus *in vitro*	[104]
	**Flavonoids** Catechins	Scavenge O_2_^−^ and Inhibited viral replication by inhibiting activities of hemagglutininn, neuraminidase and suppressing viral RNA synthesis in *in vitro* and *in vivo*	[105–107]
	Quercetin 3-rhamnoside	Inhibited viral replication by inhibiting viral mRNA synthesis	[106]
	Isoquercetin	Inhibited viral replication and pro-inflammatory cytokines	[107]
	**Antioxidant enzymes** SOD, Catalase	scavenge O_2_^−^/OH^−^ free radicals; restores redox status *in vitro* and *in vivo*; enhances recovery	[70,108–111]
	**Nrf2 inducers** Sulforaphane	antiviral activity	[39]
RSV	**Thiols** NAC	scavenge H_2_O_2_, OH^−^ free radicals, and hypochlorous acid; suppress NF-κB activation and viral replication	[51,112]
	**Polyphenols** Resveratrol	reduced IFN-γ levels associated with RSV-mediated airway inflammation and AHR; inhibit TRIF signaling pathway	[113,114]
	SOD	significantly reduced pulmonary viral titers	[73]
	SOD Mimetics	scavenge ROS and inhibit chemokine secretion *in vitro*	[8,78]
	**Nrf2 inducers** Sulforaphane BHA and tBHQ	antiviral activity in mouse; scavenge ROS by inducing expression of antioxidant enzymes and inhibit chemokine secretion *in vivo* and *in vitro*; mice treated with BHA recovered faster	[61,69] Casola A, (unpublished observation)

## References

[1] Haddad JJ (2002). Antioxidant and prooxidant mechanisms in the regulation of redox(y)-sensitive transcription factors. Cell Signal.

[2] Jones DP (2006). Redefining oxidative stress. Antioxid Redox Signal.

[3] Aruoma OI, Halliwell B, Hoey BM, Butler J (1989). The antioxidant action of N-acetylcysteine: its reaction with hydrogen peroxide, hydroxyl radical, superoxide, and hypochlorous acid. Free Radic Biol Med.

[4] Gutteridge JM, Halliwell B, Halliwell Barry, Gutteridge John MC (1992). Comments on review of Free Radicals in Biology and Medicine. Free Radic Biol Med.

[5] Rahman I (2002). Oxidative stress, transcription factors and chromatin remodelling in lung inflammation. Biochem Pharmacol.

[6] Kohen R, Nyska A (2002). Oxidation of biological systems: oxidative stress phenomena, antioxidants, redox reactions, and methods for their quantification. Toxicol Pathol.

[7] Hayes JD, McMahon M (2001). Molecular basis for the contribution of the antioxidant responsive element to cancer chemoprevention. Cancer Lett.

[8] Hosakote YM, Liu T, Castro SM, Garofalo RP, Casola A (2009). Respiratory syncytial virus induces oxidative stress by modulating antioxidant enzymes. Am J Respir Cell Mol Biol.

[9] Sharma S, Grobe AC, Wiseman DA, Kumar S, Englaish M (2007). Lung antioxidant enzymes are regulated by development and increased pulmonary blood flow. Am J Physiol Lung Cell Mol Physiol.

[10] Allen RG, Tresini M (2000). Oxidative stress and gene regulation. Free Radic Biol Med.

[11] Hosakote YM, Jantzi PD, Esham DL, Spratt H, Kurosky A (2011). Viral-mediated inhibition of antioxidant enzymes contributes to the pathogenesis of severe respiratory syncytial virus bronchiolitis. Am J Respir Crit Care Med.

[12] Valko M, Leibfritz D, Moncol J, Cronin MT, Mazur M (2007). Free radicals and antioxidants in normal physiological functions and human disease. Int J Biochem Cell Biol.

[13] Lipinski B (2011). Hydroxyl radical and its scavengers in health and disease. Oxid Med Cell Longev.

[14] Itoh K, Tong KI, Yamamoto M (2004). Molecular mechanism activating Nrf2-Keap1 pathway in regulation of adaptive response to electrophiles. Free Radic Biol Med.

[15] Osburn WO, Wakabayashi N, Misra V, Nilles T, Biswal S (2006). Nrf2 regulates an adaptive response protecting against oxidative damage following diquat-mediated formation of superoxide anion. Arch Biochem Biophys.

[16] Jaiswal AK (2004). Nrf2 signaling in coordinated activation of antioxidant gene expression. Free Radic Biol Med.

[17] Li W, Kong AN (2009). Molecular mechanisms of Nrf2-mediated antioxidant response. Mol Carcinog.

[18] Itoh K, Wakabayashi N, Katoh Y, Ishii T, Igarashi K (1999). Keap1 represses nuclear activation of antioxidant responsive elements by Nrf2 through binding to the amino-terminal Neh2 domain. Genes Dev.

[19] Wakabayashi N, Dinkova-Kostova AT, Holtzclaw WD, Kang MI, Kobayashi A (2004). Protection against electrophile and oxidant stress by induction of the phase 2 response: fate of cysteines of the Keap1 sensor modified by inducers. Proc Natl Acad Sci USA.

[20] Zhang DD, Hannink M (2003). Distinct cysteine residues in Keap1 are required for Keap1-dependent ubiquitination of Nrf2 and for stabilization of Nrf2 by chemopreventive agents and oxidative stress. Mol Cell Biol.

[21] Kaspar JW, Niture SK, Jaiswal AK (2009). Nrf2:INrf2 (Keap1) signaling in oxidative stress. Free Radic Biol Med.

[22] Baird L, Dinkova-Kostova AT (2011). The cytoprotective role of the Keap1-Nrf2 pathway. Arch Toxicol.

[23] Bloom DA, Jaiswal AK (2003). Phosphorylation of Nrf2 at Ser40 by protein kinase C in response to antioxidants leads to the release of Nrf2 from INrf2, but is not required for Nrf2 stabilization/accumulation in the nucleus and transcriptional activation of antioxidant response element-mediated NAD(P) H:quinone oxidoreductase-1 gene expression. J Biol Chem.

[24] Hybertson BM, Gao B, Bose SK, McCord JM (2011). Oxidative stress in health and disease: the therapeutic potential of Nrf2 activation. Mol Aspects Med.

[25] Boutten A, Goven D, Artaud-Macari E, Boczkowski J, Bonay M (2011). NRF2 targeting: a promising therapeutic strategy in chronic obstructive pulmonary disease. Trends Mol Med.

[26] Fitzpatrick AM, Stephenson ST, Hadley GR, Burwell L, Penugonda M (2011). Thiol redox disturbances in children with severe asthma are associated with posttranslational modification of the transcription factor nuclear factor (erythroid-derived 2)-like 2. J Allergy Clin Immunol.

[27] Cho HY, Kleeberger SR (2010). Nrf2 protects against airway disorders. Toxicol Appl Pharmacol.

[28] Ciencewicki J, Trivedi S, Kleeberger SR (2008). Oxidants and the pathogenesis of lung diseases. J Allergy Clin Immunol.

[29] Lau A, Villeneuve NF, Sun Z, Wong PK, Zhang DD (2008). Dual roles of Nrf2 in cancer. Pharmacol Res.

[30] Kensler TW, Wakabayashi N, Biswal S (2007). Cell survival responses to environmental stresses via the Keap1-Nrf2-ARE pathway. Annu Rev Pharmacol Toxicol.

[31] Tanaka N, Ikeda Y, Ohta Y, Deguchi K, Tian F (2011). Expression of Keap1-Nrf2 system and antioxidative proteins in mouse brain after transient middle cerebral artery occlusion. Brain Res.

[32] Zhu H, Itoh K, Yamamoto M, Zweier JL, Li Y (2005). Role of Nrf2 signaling in regulation of antioxidants and phase 2 enzymes in cardiac fibroblasts: protection against reactive oxygen and nitrogen species-induced cell injury. FEBS Lett.

[33] Gao L, Mann GE (2009). Vascular NAD(P)H oxidase activation in diabetes: a double-edged sword in redox signalling. Cardiovasc Res.

[34] Handa JT (2012). How does the macula protect itself from oxidative stress?. Mol Aspects Med.

[35] Miller CJ, Gounder SS, Kannan S, Goutam K, Muthusamy VR (2012). Disruption of Nrf2/ARE signaling impairs antioxidant mechanisms and promotes cell degradation pathways in aged skeletal muscle. Biochim Biophys Acta.

[36] Zhang HS, Li HY, Zhou Y, Wu MR, Zhou HS (2009). Nrf2 is involved in inhibiting Tat-induced HIV-1 long terminal repeat transactivation. Free Radic Biol Med.

[37] Cho IJ, Ki SH, Brooks C, Kim SG (2009). Role of hepatitis B virus X repression of C/EBPbeta activity in the down-regulation of glutathione S-transferase A2 gene: implications in other phase II detoxifying enzyme expression. Xenobiotica.

[38] Burdette D, Olivarez M, Waris G (2010). Activation of transcription factor Nrf2 by hepatitis C virus induces the cell-survival pathway. J Gen Virol.

[39] Kesic MJ, Simmons SO, Bauer R, Jaspers I (2011). Nrf2 expression modifies influenza A entry and replication in nasal epithelial cells. Free Radic Biol Med.

[40] Hall CB, Weinberg GA, Iwane MK, Blumkin AK, Edwards KM (2009). The burden of respiratory syncytial virus infection in young children. N Engl J Med.

[41] Shay DK, Holman RC, Newman RD, Liu LL, Stout JW (1999). Bronchiolitis-associated hospitalizations among US children, 1980–1996. JAMA.

[42] Pelletier AJ, Mansbach JM, Camargo CA (2006). Direct medical costs of bronchiolitis hospitalizations in the United States. Pediatrics.

[43] Nair H, Nokes DJ, Gessner BD, Dherani M, Madhi SA (2010). Global burden of acute lower respiratory infections due to respiratory syncytial virus in young children: a systematic review and meta-analysis. Lancet.

[44] Falsey AR (2007). Respiratory syncytial virus infection in adults. Semin Respir Crit Care Med.

[45] Han LL, Alexander JP, Anderson LJ (1999). Respiratory syncytial virus pneumonia among the elderly: an assessment of disease burden. J Infect Dis.

[46] Torrence PF, Powell LD (2002). The quest for an efficacious antiviral for respiratory syncytial virus. Anti vir Chem Chemother.

[47] Garofalo RP, Kolli D, Casola A (2013). Respiratory syncytial virus infection: mechanisms of redox control and novel therapeutic opportunities. Antioxid Redox Signal.

[48] Djordjević VB (2004). Free radicals in cell biology. Int Rev Cytol.

[49] Kooy NW, Royall JA, Ischiropoulos H (1997). Oxidation of 2′,7′-dichlorofluorescin by peroxynitrite. Free Radic Res.

[50] Marchesi E, Rota C, Fann YC, Chignell CF, Mason RP (1999). Photoreduction of the fluorescent dye 2′-7′-dichlorofluorescein: a spin trapping and direct electron spin resonance study with implications for oxidative stress measurements. Free Radic Biol Med.

[51] Casola A, Burger N, Liu T, Jamaluddin M, Brasier AR (2001). Oxidant tone regulates RANTES gene transcription in airway epithelial cells infected with Respiratory Syncytial Virus: role in viral-induced Interferon Regulatory Factor activation. J Biol Chem.

[52] Jamaluddin M, Tian B, Boldogh I, Garofalo RP, Brasier AR (2009). Respiratory syncytial virus infection induces a reactive oxygen species-MSK1-phospho-Ser-276 Rel A pathway required for cytokine expression. J Virol.

[53] Faden H, Kaul TN, Ogra PL (1983). Activation of oxidative and arachidonic acid metabolism in neutrophils by respiratory syncytial virus antibody complexes: possible role in disease. J Infect Dis.

[54] Garofalo R, Kimpen JL, Welliver RC, Ogra PL (1992). Eosinophil degranulation in the respiratory tract during naturally acquired respiratory syncytial virus infection. J Pediatrics.

[55] Indukuri H, Castro SM, Liao SM, Feeney LA, Dorsch M (2006). Ikkepsilon regulates viral-induced interferon regulatory factor-3 activation via a redox-sensitive pathway. Virology.

[56] Liu T, Castro S, Brasier AR, Jamaluddin M, Garofalo RP (2004). Reactive oxygen species mediate virus-induced STAT activation: role of tyrosine phosphatases. J Biol Chem.

[57] Fink K, Duval A, Martel A, Soucy-Faulkner A, Grandvaux N (2008). Dual role of NOX2 in respiratory syncytial virus- and sendai virus-induced activation of NF-kappaB in airway epithelial cells. J Immunol.

[58] Soucy-Faulkner A, Mukawera E, Fink K, Martel A, Jouan L (2010). Requirement of NOX2 and reactive oxygen species for efficient RIG-I-mediated antiviral response through regulation of MAVS expression. PLoS Pathog.

[59] Comstock AT, Ganesan S, Chattoraj A, Faris AN, Margolis BL (2011). Rhinovirus-induced barrier dysfunction in polarized airway epithelial cells is mediated by NADPH oxidase 1. J Virol.

[60] Vlahos R, Stambas J, Bozinovski S, Broughton BR, Drummond GR (2011). Inhibition of Nox2 oxidase activity ameliorates influenza A virus-induced lung inflammation. PLoS Pathog.

[61] Castro SM, Guerrero-Plata A, Suarez-Real G, Adegboyega PA, Colasurdo GN (2006). Antioxidant treatment ameliorates respiratory syncytial virus-induced disease and lung inflammation. Am J Respir Crit Care Med.

[62] Broor S, Bharaj P, Chahar HS (2008). Human metapneumovirus: a new respiratory pathogen. J Biosci.

[63] Bao X, Sinha M, Liu T, Hong C, Luxon BA (2008). Identification of human metapneumovirus-induced gene networks in airway epithelial cells by microarray analysis. Virology.

[64] Jacoby DB, Choi AM (1994). Influenza virus induces expression of antioxidant genes in human epithelial cells. Free Radic Biol Med.

[65] Choi AM, Knobil K, Otterbein SL, Eastman DA, Jacoby DB (1996). Oxidant stress responses in influenza virus pneumonia: gene expression and transcription factor activation. Am J Physiol.

[66] Kumar P, Khanna M, Srivastava V, Tyagi YK, Raj HG (2005). Effect of quercetin supplementation on lung antioxidants after experimental influenza virus infection. Exp Lung Res.

[67] Wakabayashi N, Slocum SL, Skoko JJ, Shin S, Kensler TW (2010). When NRF2 talks, who’s listening?. Antioxid Redox Signal.

[68] Dhakshinamoorthy S, Jain AK, Bloom DA, Jaiswal AK (2005). Bach1 competes with Nrf2 leading to negative regulation of the antioxidant response element (ARE)-mediated NAD(P)H:quinoneoxidoreductase 1 gene expression and induction in response to antioxidants. J Biol Chem.

[69] Cho HY, Imani F, Miller-DeGraff L, Walters D, Melendi GA (2009). Antiviral activity of Nrf2 in a murine model of respiratory syncytial virus disease. Am J Respir Crit Care Med.

[70] Yageta Y, Ishii Y, Morishima Y, Masuko H, Ano S (2011). Role of Nrf2 in host defense against influenza virus in cigarette smoke-exposed mice. J Virol.

[71] Kosmider B, Messier EM, Janssen WJ, Nahreini P, Wang J (2012). Nrf2 protects human alveolar epithelial cells against injury induced by influenza A virus. Respir Res.

[72] Uchide N, Toyoda H (2011). Antioxidant therapy as a potential approach to severe influenza-associated complications. Molecules.

[73] Wyde PR, Moore DK, Pimentel DM, Gilbert BE, Nimrod R (1996). Recombinant superoxide dismutase (SOD) administered by aerosol inhibits respiratory syncytial virus infection in cotton rats. Antiviral Res.

[74] Batinić-Haberle I, Rebouças JS, Spasojević I (2010). Superoxide dismutase mimics: chemistry, pharmacology, and therapeutic potential. Antioxid Redox Signal.

[75] Baudry M, Etienne S, Bruce A, Palucki M, Jacobsen E (1993). Salen-manganese complexes are superoxide dismutase-mimics. Biochem Biophys Res Commun.

[76] Doctrow SR, Huffman K, Marcus CB, Tocco G, Malfroy E (2002). Salen-manganese complexes as catalytic scavengers of hydrogen peroxide and cytoprotective agents: structure-activity relationship studies. J Med Chem.

[77] Gonzalez PK, Zhuang J, Doctrow SR, Malfroy B, Benson PF (1995). EUK-8, a synthetic superoxide dismutase and catalase mimetic, ameliorates acute lung injury in endotoxemic swine. J Pharmacol Exp Ther.

[78] Hosakote YM, Komaravelli N, Mautemps N, Liu T, Garofalo RP (2012). Antioxidant mimetics modulate oxidative stress and cellular signaling in airway epithelial cells infected with respiratory syncytial virus. Am J Physiol Lung Cell Mol Physiol.

[79] Liby KT, Yore MM, Sporn MB (2007). Triterpenoids and rexinoids as multifunctional agents for the prevention and treatment of cancer. Nat Rev Cancer.

[80] Sporn MB, Liby KT, Yore MM, Fu L, Lopchuk JM (2011). New synthetic triterpenoids: potent agents for prevention and treatment of tissue injury caused by inflammatory and oxidative stress. J Nat Prod.

[81] Surh YJ, Kundu JK, Na HK (2008). Nrf2 as a master redox switch in turning on the cellular signaling involved in the induction of cytoprotective genes by some chemopreventive phytochemicals. Planta Med.

[82] Hur W, Gray NS (2011). Small molecule modulators of antioxidant response pathway. Curr Opin Chem Biol.

[83] Keum YS, Han YH, Liew C, Kim JH, Xu C (2006). Induction of heme oxygenase-1 (HO-1) and NAD[P]H: quinoneoxidoreductase 1 (NQO1) by a phenolic antioxidant, butylatedhydroxyanisole (BHA) and its metabolite, tert-butylhydroquinone (tBHQ) in primary-cultured human and rat hepatocytes. Pharm Res.

[84] Papi A, Papadopoulos NG, Stanciu LA, Bellettato CM, Pinamonti S (2002). Reducing agents inhibit rhinovirus-induced up-regulation of the rhinovirus receptor intercellular adhesion molecule-1 (ICAM-1) in respiratory epithelial cells. FASEB J.

[85] Biagioli MC, Kaul P, Singh I, Turner RB (1999). The role of oxidative stress in rhinovirus induced elaboration of IL-8 by respiratory epithelial cells. Free Radic Biol Med.

[86] Kaul P, Biagioli MC, Singh I, Turner RB (2000). Rhinovirus-induced oxidative stress and interleukin-8 elaboration involves p47-phox but is independent of attachment to intercellular adhesion molecule-1 and viral replication. J Infect Dis.

[87] Papi A, Contoli M, Gasparini P, Bristot L, Edwards MR (2008). Role of xanthine oxidase activation and reduced glutathione depletion in rhinovirus induction of inflammation in respiratory epithelial cells. J Biol Chem.

[88] Akaike T, Ando M, Oda T, Doi T, Ijiri S (1990). Dependence on O2-generation by xanthine oxidase of pathogenesis of influenza virus infection in mice. J Clin Invest.

[89] Akaike T, Noguchi Y, Ijiri S, Setoguchi K, Suga M (1996). Pathogenesis of influenza virus-induced pneumonia: involvement of both nitric oxide and oxygen radicals. Proc Natl Acad Sci U S A.

[90] Zaki MH, Akuta T, Akaike T (2005). Nitric oxide-induced nitrative stress involved in microbial pathogenesis. J Pharmacol Sci.

[91] Nencioni L, Sgarbanti R, De Chiara G, Garaci E, Palamara AT (2007). Influenza virus and redox mediated cell signaling: a complex network of virus/host interaction. New Microbiol.

[92] Tsutsumi H, Takeuchi R, Ohsaki M, Seki K, Chiba S (1999). Respiratory syncytial virus infection of human respiratory epithelial cells enhances inducible nitric oxide synthase gene expression. J Leukoc Biol.

[93] Krenn BM, Holzer B, Gaudernak E, Triendl A, van Kuppeveld FJ (2005). Inhibition of polyprotein processing and RNA replication of human rhinovirus by pyrrolidinedithiocarbamate involves metal ions. J Virol.

[94] Knobil K, Choi AM, Weigand GW, Jacoby DB (1998). Role of oxidants in influenza virus-induced gene expression. Am J Physiol.

[95] Kelly GS (1998). Clinical applications of N-acetylcysteine. Altern Med Rev.

[96] Garozzo A, Tempera G, Ungheri D, Timpanaro R, Castro A (2007). N-acetylcysteine synergizes with oseltamivir in protecting mice from lethal influenza infection. Int J Immunopathol Pharmacol.

[97] Cai J, Chen Y, Seth S, Furukawa S, Compans RW (2003). Inhibition of influenza infection by glutathione. Free Radic Biol Med.

[98] Fraternale A, Paoletti MF, Casabianca A, Nencioni L, Garaci E (2009). GSH and analogs in antiviral therapy. Mol Aspects Med.

[99] Uchide N, Ohyama K, Bessho T, Toyoda H (2005). Inhibition of influenza-virus-induced apoptosis in chorion cells of human fetal membranes by nordihydroguaiaretic Acid. Intervirology.

[100] Miyamoto D, Kusagaya Y, Endo N, Sometani A, Takeo S (1998). Thujaplicin-copper chelates inhibit replication of human influenza viruses. Antiviral Res.

[101] Palamara AT, Nencioni L, Aquilano K, De Chiara G, Hernandez L (2005). Inhibition of influenza A virus replication by resveratrol. J Infect Dis.

[102] Huang YL, Loke SH, Hsu CC, Chiou WF (2008). (+)-Vitisin A inhibits influenza A virus-induced RANTES production in A549 alveolar epithelial cells through interference with Akt and STAT1 phosphorylation. Planta Med.

[103] Yang B, Yao DF, Ohuchi M, Ide M, Yano M (2002). Ambroxol suppresses influenza-virus proliferation in the mouse airway by increasing antiviral factor levels. Eur Respir J.

[104] Furuya A, Uozaki M, Yamasaki H, Arakawa T, Arita M (2008). Antiviral effects of ascorbic and dehydroascorbic acids in vitro. Int J Mol Med.

[105] Song JM, Lee KH, Seong BL (2005). Antiviral effect of catechins in green tea on influenza virus. Antiviral Res.

[106] Choi HJ, Song JH, Park KS, Kwon DH (2009). Inhibitory effects of quercetin 3-rhamnoside on influenza A virus replication. Eur J Pharm Sci.

[107] Kim Y, Narayanan S, Chang KO (2010). Inhibition of influenza virus replication by plant-derived isoquercetin. Antiviral Res.

[108] Oda T, Akaike T, Hamamoto T, Suzuki F, Hirano T (1989). Oxygen radicals in influenza-induced pathogenesis and treatment with pyran polymer-conjugated SOD. Science.

[109] Sidwell RW, Huffman JH, Bailey KW, Wong MH, Nimrod A (1996). Inhibitory effects of recombinant manganese superoxide dismutase on influenza virus infections in mice. Antimicrob Agents Chemother.

[110] Suliman HB, Ryan LK, Bishop L, Folz RJ (2001). Prevention of influenza-induced lung injury in mice overexpressing extracellular superoxide dismutase. Am J Physiol Lung Cell Mol Physiol.

[111] Shi XL, Shi ZH, Huang H, Zhu HG, Zhou P (2010). Therapeutic effect of recombinant human catalase on H1N1 influenza-induced pneumonia in mice. Inflammation.

[112] Mata M, Morcillo E, Gimeno C, Cortijo J (2011). N-acetyl-l-cysteine (NAC) inhibit mucin synthesis and pro-inflammatory mediators in alveolar type II epithelial cells infected with influenza virus A and B and with respiratory syncytial virus (RSV). Biochem Pharmacol.

[113] Zang N, Xie X, Deng Y, Wu S, Wang L (2011). Resveratrol-mediated gamma interferon reduction prevents airway inflammation and airway hyperresponsiveness in respiratory syncytial virus-infected immunocompromised mice. J Virol.

[114] Drago L, Nicola L, Ossola F, De Vecchi E (2008). In vitro antiviral activity of resveratrol against respiratory viruses. J Chemother.

